# Dynamic brain states underlying advanced concentrative absorption meditation: A 7-T fMRI-intensive case study

**DOI:** 10.1162/netn_a_00432

**Published:** 2025-03-03

**Authors:** Isaac N. Treves, Winson F. Z. Yang, Terje Sparby, Matthew D. Sacchet

**Affiliations:** Meditation Research Program, Department of Psychiatry, Massachusetts General Hospital, Harvard Medical School, Boston, MA, USA; Athinoula A. Martinos Center for Biomedical Imaging, Department of Radiology, Massachusetts General Hospital, Harvard Medical School, Boston, MA, USA; Department of Brain and Cognitive Sciences, Massachusetts Institute of Technology, Cambridge, MA, USA; Rudolf Steiner University College, Oslo, Norway; Department of Psychology and Psychotherapy, Witten/Herdecke University, Witten, Germany; Integrated Curriculum for Anthroposophic Psychology, Witten/Herdecke University, Witten, Germany

**Keywords:** Advanced meditation, Dynamic brain states, DFC, fMRI, Consciousness

## Abstract

Advanced meditation consists of states and stages of practice that unfold with mastery and time. Dynamic functional connectivity (DFC) analysis of fMRI could identify brain states underlying advanced meditation. We conducted an intensive DFC case study of a meditator who completed 27 runs of *jhāna* advanced absorptive concentration meditation (ACAM-J), concurrently with 7-T fMRI and phenomenological reporting. We identified three brain states that marked differences between ACAM-J and nonmeditative control conditions. These states were characterized as a DMN-anticorrelated brain state, a hyperconnected brain state, and a sparsely connected brain state. Our analyses indicate higher prevalence of the DMN-anticorrelated brain state during ACAM-J than control states, and the prevalence increased significantly with deeper ACAM-J states. The hyperconnected brain state was also more common during ACAM-J and was characterized by elevated thalamocortical connectivity and somatomotor network connectivity. The hyperconnected brain state significantly decreased over the course of ACAM-J, associating with self-reports of wider attention and diminished physical sensations. This brain state may be related to sensory awareness. Advanced meditators have developed well-honed abilities to move in and out of different altered states of consciousness, and this study provides initial evidence that functional neuroimaging can objectively track their dynamics.

## Author Summary

Advanced meditation research investigates states and stages of practice that unfold with increasing mastery and time, which may include altered states of consciousness such as a diminished sense of self. In the current study, we examined a 7-T fMRI case study of *jhāna*, an advanced concentrative absorptive meditation (ACAM-J). Specifically, we examined the temporal properties of dynamic connectivity brain states that could reflect mental states and phenomena during ACAM-J. We identified two brain states that were more prevalent during ACAM-J than control conditions. One state, involving default-mode network anticorrelations with the rest of the brain, increased across ACAM-J. Another state, involving hyperconnectivity across many cortical networks, was correlated with reports of narrow attention and greater sensory awareness, as well as diminished across ACAM-J.

## INTRODUCTION

Meditation typically involves intentional attention regulation (Lutz et al., 2008; Sparby & Sacchet, 2022), which may lead to changes in states of consciousness (Josipovic, 2014; Timmermann et al., 2023); insight into the self and perception (Cooper et al., 2022; Giommi et al., 2023); and positive experiences of empathy, compassion, and joy (Desbordes et al., 2015; Hölzel et al., 2011; Woods et al., 2023). There has been substantial neuroscientific research on brain changes associated with the effects of meditation (e.g., Ganesan et al., 2022; Sezer et al., 2022). However, most research has been conducted on basic mindfulness skills (Harrington & Dunne, 2015; Kang & Whittingham, 2010; Samuel, 2015), and there is little systematic research on advanced meditation. Advanced meditation research investigates states and stages of practice that unfold with increasing mastery and time, in some cases, thousands of hours of training. Advanced meditation research expands our knowledge of human mental capacities and limits, as well as their underlying brain bases, and could inform domains including mental health, peak performance, and artificial intelligence (Sacchet et al., 2024; Wright et al., 2024). Here, we investigate the brain bases of *jhāna*, an advanced concentrative absorptive meditation (ACAM-J), with origins in Theravada Buddhism (Gunaratana, 1988).

*Jhāna* meditation, hereon ACAM-J, consists of eight progressive states of altered consciousness, classified into *form* and *formless* jhanas. Traditional accounts describe ACAM-J as a series of progressive meditative states starting with ACAM-J1 and ending with ACAM-J8. ACAM-J is often characterized by five factors: directed attention, sustained attention, emotional joy, mental ease, and sometimes single pointedness (Buddhaghosa, 2020; Sayadaw, 2008; Yang, Chowdhury, et al., 2024). Progression through the *form* jhanas entails a gradual refinement and shift of focus from these factors, which gradually diminish until only mental ease and single pointedness remain. The following *formless* jhanas are difficult to describe (e.g., “boundless consciousness”), but they involve expansive attention, total lack of spontaneous, undirected thought (Gunaratana, 1988), and diminishing of body awareness (Brasington, 2015; Sparby & Sacchet, 2024; Yang, Chowdhury, et al., 2024).

Neuroscientific investigation of ACAM-J meditation is in its infancy (Yang, Chowdhury, et al., 2024); several fMRI studies of ACAM-J, all single-case studies, have been conducted (Ganesan et al., 2024.; Hagerty et al., 2013; Yang, Sparby, et al., 2024). Studies thus far have focused on comparisons of ACAM-J with nonmeditative control conditions, as well as how measures like Regional Homogeneity (ReHo, associated with neuronal activation) of voxels and regions change over the course of ACAM-J states. Results indicated increased ReHo in sensory regions like visual cortex and systematic shifts in ReHo from anterior to posterior regions throughout the jhanas (Yang, Sparby, et al., 2024). Thus far, there has been no research on changes in network interactions (i.e., connectivity) that support ACAM-J states. It has been observed across a range of imaging modalities that brain connectivity is nonstationary and fluctuates in tandem with fluctuations in arousal, attention, and consciousness (Damaraju et al., 2020; Hutchison et al., 2013; Kucyi, 2018; Yamashita et al., 2021).

The emerging field of dynamic functional connectivity (DFC) investigates these dynamic interactions (Preti et al., 2017). One influential method of DFC is brain connectivity clustering (Allen et al., 2014). In this method, brain connectivity is assessed using correlations between brain networks or regions within segmented and overlapping time windows. Then, the connectivity matrices are clustered using *k*-means into the most distinctive, prototypical connectivity matrices called “brain states.” During meditation, these brain states may emerge and recede over time in tandem with phenomenological changes.

While there is a growing literature on brain connectivity in mindfulness and meditation (Sezer et al., 2022; Treves, Pichappan, et al., 2024), only a few studies have applied DFC to meditation, and none to advanced meditation. A study of breathing meditation in novices found that individuals occupied multiple brain states including a default mode network (DMN)-anticorrelated brain state, and dwell time in this state increased after a mindfulness intervention (Mooneyham et al., 2017). They interpreted this as related to diminished self-referential mind wandering. DFC has also been used to explore brain states related to sensory awareness in meditators (Panitz et al., 2023) and the temporal complexity of brain activity during meditation (Escrichs et al., 2019). These studies provide a proof of concept for the application of DFC to advanced meditation for the purposes of examining brain states related to sensation, attention, and self-perception, along with dynamical properties like brain state switching.

Here, we conducted a DFC analysis of an advanced ACAM-J meditator using ultrahigh-field 7-T fMRI. Case studies, while limited in generalizability, are essential tools for charting the frontiers of cognitive neuroscience (Supporting Information). DFC analysis is an effective, data-driven method for identifying state changes during meditation, which we relate to granular phenomenological reports. More generally, our analysis contributes to the growing body of research on advanced meditation.

## MATERIALS AND METHODS

### Case Study

Data were collected from one White American male advanced meditator who was 52 years old at the time of the study. The participant is a long-term meditation teacher with over 25 years of meditation experience. This participant has trained extensively in both concentration and insight meditation across various traditions, such as Thai forest tradition, Burmese Mahasi tradition, and Tibetan Buddhism. Based on their reports, we estimate a total practice amount of at least 20,000 hr. In the current study, the participant performed ACAM-J. In brief, the participant’s meditation style during scanning was most like the *sutta-jhana* as the participant reported using the breath, bodily feelings, and width of attention to enter the *form* jhanas and formless objects (i.e., space, consciousness, nothingness, neither perception nor nonperception) to enter the *formless* jhanas. The Mass General Brigham institutional review board approved the research study, and the participant gave informed consent.

### Study Procedure

#### ACAM-J.

fMRI data were collected throughout five consecutive days. Depending on the ability and comfort of the participant, each day consisted of 45–90 min of meditation concurrent with fMRI scans. We asked the participant to meditate eyes closed using their typical ACAM-J meditation sequence. The participant began with access concentration (AC), continuing through first to eighth jhana (J1–J8), and finished with the afterglow. Upon every transition from AC to J6, the participant indicated the start of a given state using a button. The participant did not indicate transitions from J6 to J7 and J7 to J8 (as the meditator indicated this would disrupt the continuation of their practice (Yang, Sparby, et al., 2024). After each run, we collected self-reports (see the Phenomenology section below). In total, we recorded 27 runs of jhana. The average duration for a complete run for this set of analyses was 8.53 min (512.01 s), and overall jhana duration increased over the course of the runs (noise-free runs, *r*(26) = 0.60, *p* = 0.001). Specific durations of each jhana are found in Supporting Information Table S1.

#### Control conditions.

We developed two control conditions for comparison with meditation states. These control conditions were designed to engage the participant in nonmeditative cognition. We did not use a resting-state control condition because experienced meditators may meditate at rest (Tang et al., 2012; Yang, Sparby, et al., 2024). The first control condition consisted of a memory control task: The participant was asked to remember the events of the past 2 weeks and silently narrate them in their mind, without moving their lips, for 8 min. In the second control task, the participant was asked to count down, mentally without moving the lips, in decreasing intervals of 5 from 10,000 for 8 min. We collected two runs of each control condition, resulting in 16 min data for each control task. For increased statistical power, we collapsed across the control conditions in analyses.

#### Phenomenology.

We implemented a neurophenomenological approach consisting of linking neural measures to first-person experience of the meditations (Lutz & Thompson, 2003). Our participant comprehensively evaluated the mental and physiological processes relevant to the experience of *jhanas* as they manifested. The phenomenological items used in this study were “(1) stability of attention, defined as the calmness and tranquility from poor to excellent stability; (2) width of attention, ranging from narrow scope like a laser to very wide like a fisheye lens; (3) intensity of jhanas, defined as how jhanic a particular jhana was; (4) quality of J2 (joy) defined as energetic sensations in the body such as tingling, lightning-like or electric sensations, and one or more waves moving through the body; (5) sensations of early narrative thought stream, described as the presence of narrative thought contents during the form jhanas; (6) sensations of late narrative thought stream, described as the presence of narrative thought contents during the formless jhanas; (7) sensations of physical sensations” (Yang, Sparby, et al., 2024, p. 4). Each item was rated on a scale from 1 to 10, with higher values indicating a stronger experience within the given item. We computed “global” or “local” measures of phenomenology based on these items. Global measures consist of calculations of averages of items resulting in overall scores for each run (27 scores), and we calculated attentional stability, intensity, width. Local measures consist of items rated for each of the jhanas (162 ratings: 27 runs consisting of J1, J2, J3, J4, J5, and J6–J8), and consisted of attention stability, intensity and width, as well as bliss, joy, and equanimity. Finally, sensations and narrative processing were separately rated for *form* and *formless* stages of the 27 runs (54 ratings).

### Neuroimaging Acquisition

Neuroimaging scans were acquired on a 7-T MRI scanner (SIEMENS MAGNETOM Terra) with a 32-channel head coil. Functional imaging was performed using a single-shot, two-dimensional echo planar imaging sequence with T2∗weighted BOLD-sensitive MRI, repetition time (TR) = 2.9 s, echo time (TE) = 30 ms, flip angle (FA) = 75°, field of view (FOV) = (189 × 255), matrix = (172 × 232), GRAPPA factor = 3, voxel size = 1.1 × 1.1 × 1.1 mm^3^, 126 slices, interslice distance = 0 mm, bandwidth = 1,540 Hz/px, echo spacing = 0.75 ms. Slice acquisitions were acquired for the whole brain, with interleaved slices, sagittal orientation, and anterior-to-posterior phase encoding. Opposite phase-encoded (i.e., posterior-to-anterior) slices with the same parameters were also acquired to perform distortion correction. Whole-brain T1-weighted structural images were acquired as follows: TR = 2.53 s, TE = 1.65 ms, inversion time = 1.1 s, FA = 7°, 0.8 mm isotropic resolution, FOV = 240 × 240, GRAPPA factor = 2, bandwidth = 1,200 Hz/Px. The participant’s physiological (i.e., heart rate using pulse oximetry and respiration using breathing bellows) signal recordings were collected throughout the scanning sessions.

### Neuroimaging Preprocessing

Preprocessing steps were carried out at the level of the session and for all runs contained in that session. We conducted fieldmap correction, despiking, physiological noise correction (Glover et al., 2000), and slice time correction in AFNI. Further, in CONN, we normalized, segmented, and applied minimal smoothing using a 2-mm full-width half-maximum kernel to allow for granular differences in the brainstem (Whitfield-Gabrieli & Nieto-Castanon, 2012).

### Neuroimaging Denoising

To denoise the fMRI data, we applied linear regression to remove the following parameters from each voxel: (a) five noise components each from minimally eroded WM and CSF (one-voxel binary erosion of voxels with values above 50% in posterior probability maps), respectively, based on aCompCor procedures; (b) 12 motion parameters (three translation, three rotation, and associated first-order derivatives); and (c) linear BOLD signal trend within session. We did not apply global signal regression, per standard CONN procedures. In a separate step after nuisance regression, data were temporally filtered with a bandpass of 0.01–0.1 Hz. Rather than removing (“scrubbing”) high motion frames, which may be nonoptimal for DFC (Allen et al., 2014), we conducted despiking after regression. Despiking applies a tangent squashing function, which is essentially linear for signal within 3 *SD*s of the mean, and exponential outside.

### Regions of Interest

We parcellated the brain using four different atlases: (a) Schaefer-400 parcellation atlas for cortical areas (Schaefer et al., 2018); (b) 62 Tian subcortex atlas for subcortical regions (Tian et al., 2020); (c) 54 Bianciardi brainstem atlas (Bianciardi et al., 2016); and (d) Multi-Domain Task Battery (MDTB) functional cerebellar atlas (King et al., 2019). Before analysis, we reduced the timecourses to 49 networks and regions of interest defined by our previous study (Yang, Sparby, et al., 2024). This targeted analysis consisted of 33 cortical networks, 10 subcortical regions, five brainstem regions, and the cerebellum (Supporting Information Table S3, Supporting Information File S1). This step was taken based on previous evidence that these brain regions were modulated by jhana, and because it is well-established that clustering methods benefit from reduced dimensionality (Bouveyron & Brunet-Saumard, 2014). We conducted a secondary analysis with all 526 regions to evaluate the specificity of our findings.

### Static Functional Connectivity Analysis

We compared functional connectivity values computed as correlations between timecourses across the entire run for the 49 regions/networks. Correlations were fisher’s *z* transformed. We compared jhana runs with the average of control runs descriptively and through the use of one-sample *t* tests (compared with average control value) on pairwise connectivity using Bonferroni corrections.

### Dynamic Functional Connectivity Analysis

After extracting timecourses from the 49 regions of interest, we conducted windowed correlation analyses using GIFT toolbox scripts (Correa et al., 2005), utilizing tapered Gaussian windows of length 16 TRs (the *icatb_compute_dfnc* function). The window length of 16 TRs (46.4 s) was chosen to reduce overlap between successive jhana states, and research has shown that 30–60 s is sufficient for robust estimation of connectivity states (Damaraju et al., 2020). We then concatenated connectivity matrices across control runs and jhana runs to find the number of optimized clusters using k-means clustering technique (*icatb_optimal_clusters*). After optimizing the number of clusters, we then conducted k-means clustering again, but with a fixed number of clusters to derive the connectivity states (*icatb_kmeans_clustering*). Post visual inspection, states that showed noise (highly variable correlations in adjacent pairwise connections) and were present only in one jhana run were removed.

To visualize the connectivity patterns of the states, we conducted a graph-based method using the *qgraph* package in R (Epskamp et al., 2012). This method clusters networks/ROIs based on similarity in correlations. We used an absolute value threshold of 0.3 to display the connections.

The dynamic measures calculated were: proportion of time in each brain state, the average dwell time in each state, the number of episodes in a brain state, and the total proportion of switches relative to TRs in a run. In addition, we calculated transition matrices between the brain connectivity states for jhana and control.

#### Clustering trajectories.

We further explored clusters of DFC state trajectories using k-modes (Huang, 1998), given the variety of dynamics and phenomenology across runs in this meditator. K-modes clustering is a clustering method tailored for categorical data, which identifies the dominant category for each observation at every timepoint. We extracted the most prevalent brain state from each jhana stage and run, and then applied K-modes using the elbow criterion to determine the optimal number of trajectory clusters. We then applied this method with the optimal number of clusters to group jhana runs based on trajectories.

### Statistical Analysis

#### ACAM-J versus control.

We compared static connectivity between control and ACAM-J using unpaired *t* tests with Bonferroni corrections over the pairwise edges. We also compared dynamic measures between the control and jhana (this is an advantage of clustering using both conditions). As there are different numbers of control versus jhana runs, we averaged across control runs to get a reference value for each static connectivity edge and dynamic measure. Then, single sample *t* tests were conducted between the set of ACAM-J run static edges and dynamic values versus average control values.

#### ACAM-J trajectories.

Mixed-effects logistic regressions using the *glmer* function in R were used to predict the likelihood of presence of a specific brain state (e.g., *BrainState1_Present*) at each jhana state (J1, J2, J3, J4, J5, J6–8), through the course of ACAM-J meditation. The jhana state was set as the fixed effect while each run was set as the random intercept for jhana run (*glmer*(*BrainState1_Present* ∼ (*1*∣*JhanaRun* + *JhanaState*, *family* = “*binomial*”)). This controls for possible differences in brain states over runs. In addition to predicting brain states using jhana state, we used jhana category (*form* vs. *formless*).

#### Jhana phenomenology.

We correlated global and local phenomenology ratings with dynamic measures of the brain states. Multiple comparisons correction was not used to correct for multiple testing given the nonindependence of the brain states (decreases in one state result in increases in the others). We also explored canonical correlation analyses between global phenomenology ratings and brain state proportions to assess multivariate associations.

## RESULTS

### Static Functional Connectivity (SFC)

**Figure 1.**
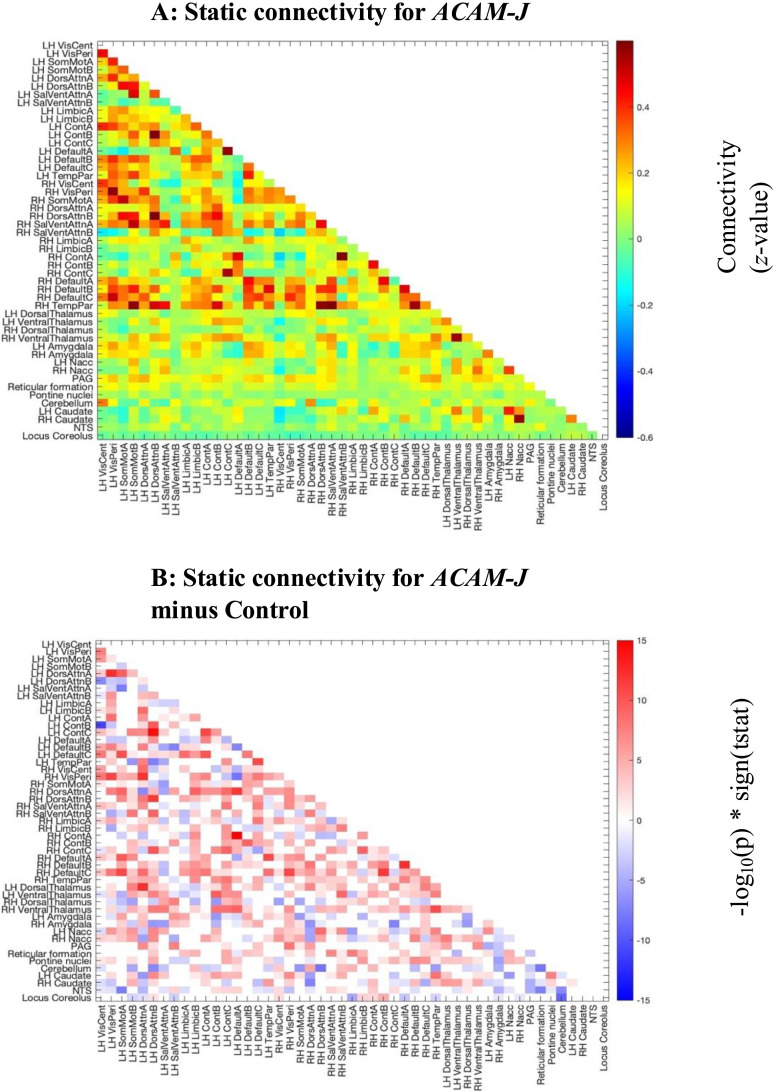
SFC during ACAM-J. (A) The *z*-scored connectivity values are plotted for the average of all 26 *jhana* runs. (B) The results of a one-sample *t* test for *jhana* SFC minus control SFC. The color indicates how significant the difference was; red colors represent positive differences and blue colors represent negative differences and nonsignificant pairwise connections are in white.

The average SFC for ACAM-J is displayed in Figure 1A. As assessed by *t* tests with strict Bonferroni correction, SFC between cortical networks was higher during ACAM-J than during nonmeditative control conditions (Figure 1B). SFC was lower during ACAM-J between brainstem regions like the nucleus tractus solitarii and locus coeruleus, and the rest of the brain (Figure 1B).

### Dynamic Functional Connectivity (DFC)

**Figure 2.**
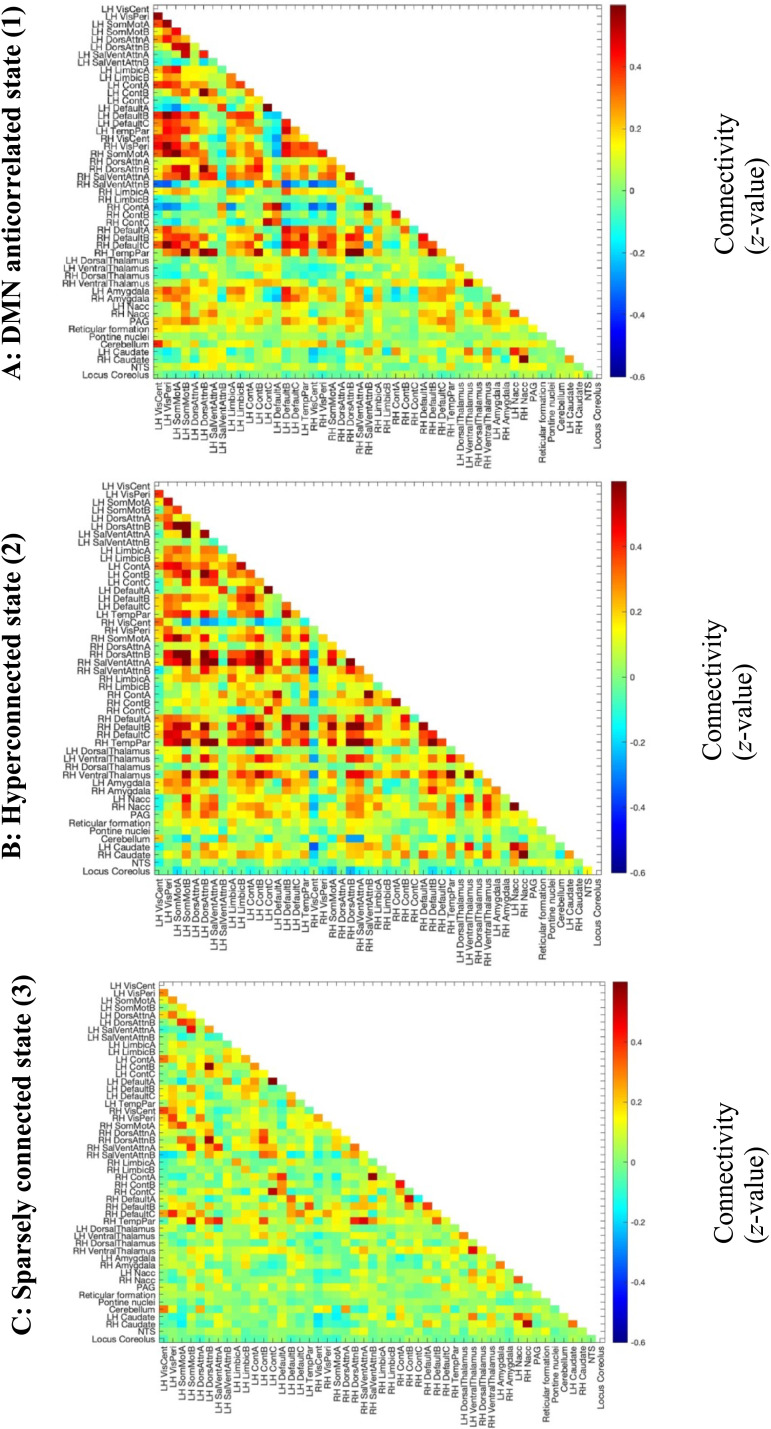
Dynamic functional connectivity brain state clustering. (A) Brain State 1, also called DMN-anticorrelated brain state. (B) Brain State 2, also called hyperconnected brain state. (C) Brain State 3, also called sparsely connected brain state.

The optimal number of clusters as derived by the elbow criterion was five. Two of the five clusters were removed because they were only present in ACAM-J run 27 (and run 27 only contained those clusters) and showed highly unstructured and noise-like connectivity. The remaining clusters are plotted in Figure 2. In brief, Brain State 1 involves anticorrelations between DMN and the rest of the brain, Brain State 2 involves hyperconnectivity across cortex and subcortex including thalamus, and Brain State 3 is sparsely connected. Hereafter, we focus on Brain States 1 and 2 as they showed changes due to jhana and relationships to phenomenology.

Brain State 1 involves high cortical connectivity, with notable anticorrelations (negative correlations) between left DMN A and the rest of the brain, as well as between right control network A and right salience network B and the rest of the brain. Much of the subcortical areas and brainstem were low in connectivity, with the exception of the periaqueductal grey and amygdala, which were positively correlated with cortical areas. There was some positive connectivity within striatal regions. See Supporting Information Figure S1 for network graph visualization. We henceforth call this State 1, or the DMN-anticorrelated state.

Brain State 2, on the other hand, showed negative connectivity between the right hemisphere visual central network and the rest of the brain, but no other anticorrelations of note. See Supporting Information Figure S2 for network graph visualization. Connectivity was elevated throughout, with the highest correlations observed between the left and right DMN, dorsal attention (DAN), ventral attention (VAN), and somatomotor (SMN) networks, and the rest of the brain. The right hemisphere control network was not positively correlated with the rest of the brain. Notably, in comparison with Brain State 1, the thalamus and striatal regions showed correlations with cortical regions (Supporting Information Figure S3). In addition, the cerebellum was positively correlated with the dorsal attention network.

### Dynamic Measures of Brain States

We compared dynamic measures of the brain states for ACAM-J versus control conditions. Differences were widespread (Table 1). During ACAM-J, the practitioner spent more time in states 1 and 2—corresponding to marginally longer dwell times (respectively, *p*_*FDR*_ = 0.046, 0.079) and more frequent episodes (*p*_*FDR*_ < 0.01). During control conditions, the practitioner spent significantly more time in Brain State 3 (*p*_*FDR*_ < 0.001), consisting of a very large increase in average dwell time in the state (control *M* = 35.20 TRs, ACAM-J *M* = 20.79 TRs). ACAM-J runs contained significantly more switches between brain states than control conditions, with no difference between *form* and *formless* (*p* = 0.14).

**Table 1.** Comparison of dynamic measures between ACAM-J and control

**Measure**	**ACAM-J**	**Control**	**Difference**	***p*_*FDR*_**
State 1 proportion	0.31	0.19	0.12	0.0067**
State 2 proportion	0.27	0.16	0.11	0.0067**
State 3 proportion	0.42	0.65	−0.23	<0.001***
State 1 episodes	3.15	2.00	1.15	0.0019**
State 2 episodes	2.73	2.00	0.73	0.0081**
State 3 episodes	3.27	3.50	−0.23	0.3451
State 1 dwell time	14.66	10.69	3.98	0.0463*
State 2 dwell time	15.91	12.33	3.57	0.0798
State 3 dwell time	20.79	35.20	−14.42	<0.001***
Proportion switch	0.05	0.04	0.01	0.0081**

Proportion reflects the proportion of TRs spent in a given state. Episodes refers to the number of unique periods spent in a given state. Dwell time reflects the average time spent in a brain state for a single episode. Proportion switch reflects how often (what proportion of TRs) involved a switch between brain states.

* *p* < 0.05.

** *p* < 0.01.

*** *p* < 0.001.

We also examined the transition matrices between brain states, which indicated differences in the dynamics between ACAM-J and control (Supporting Information Figure S4). Full results are described in the supplemental results.

### Trajectories of Brain States Over ACAM-J

**Figure 3.**
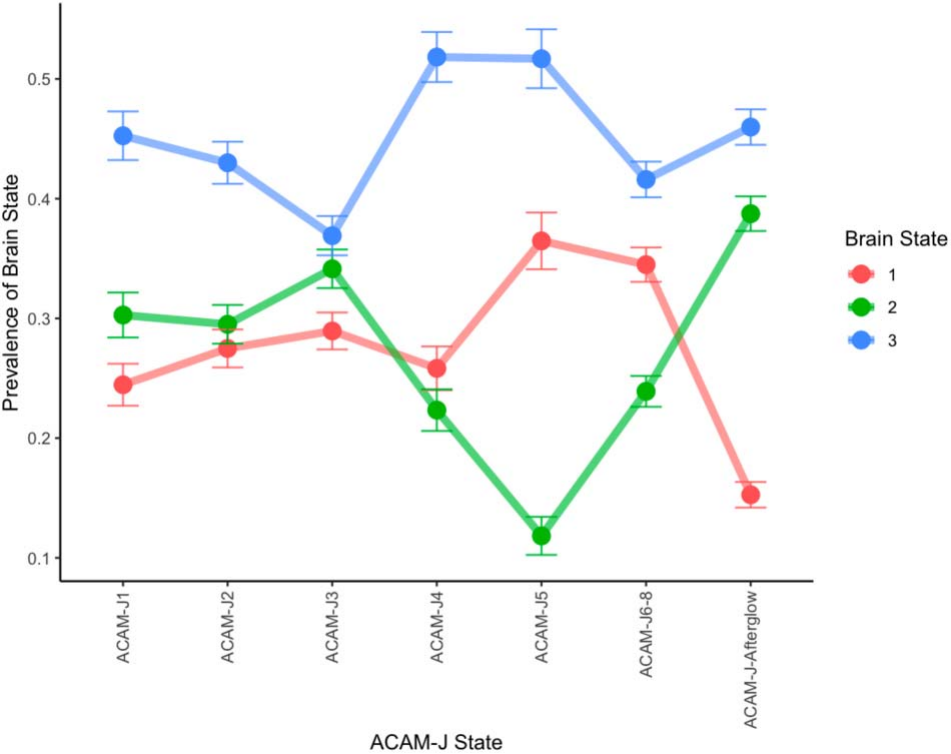
Average prevalence of each brain state during each ACAM-J state. Standard errors are plotted for each jhana state and brain state. Afterglow refers to the period after the jhanas are completed.

**Figure 4.**
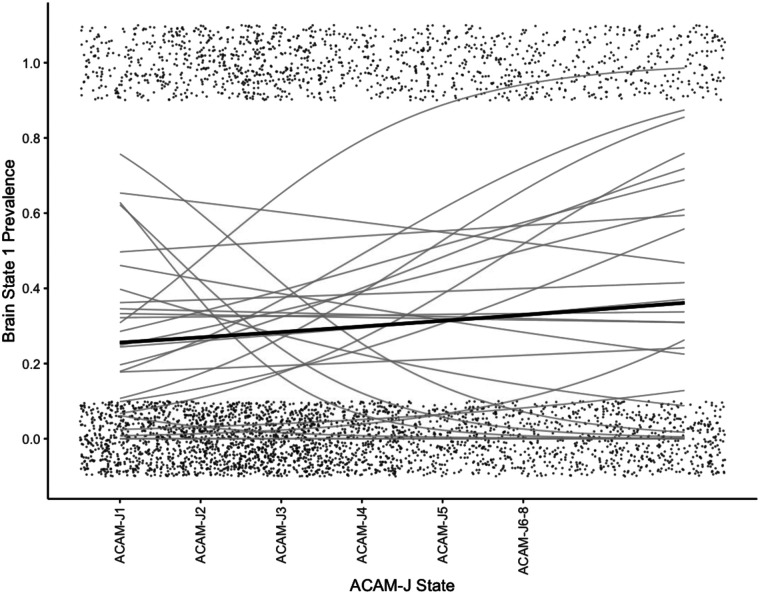
Logistic model fits for Brain State 1 prevalence during ACAM-J. ACAM-J states are reflected on the *x*-axis while the prevalence of the brain state is reflected on the *y*-axis. Points reflect the presence of brain state (0 = not present, 1 = present), and are jittered for ease of viewing. Points for ACAM-J6–J8 are extended to match durations of previous ACAM-J states. The gray lines reflect logistic fits for each ACAM-J run, and the black line is the mean, with gray standard error shading. Afterglow is not plotted because main logistic models exclude afterglow.

**Figure 5.**
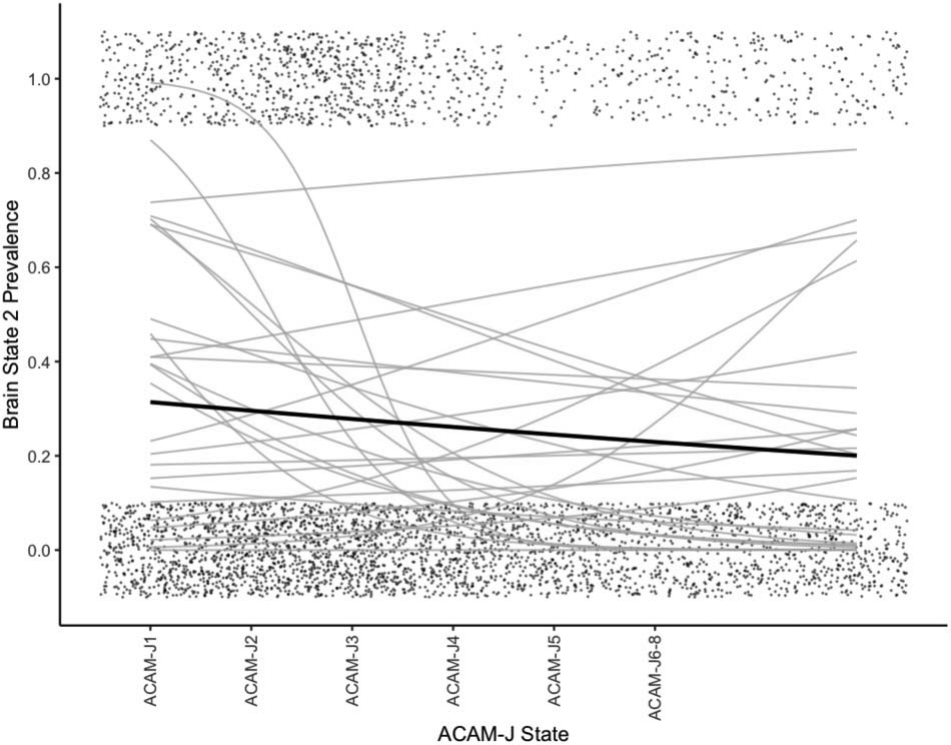
Logistic model fits for Brain State 2 prevalence during ACAM-J. ACAM-J states are reflected on the *x*-axis while the prevalence of the brain state is reflected on the *y*-axis. Points reflect the presence of brain state (0 = not present, 1 = present), and are jittered for ease of viewing. Points for ACAM-J6–8 are extended to match durations of previous ACAM-J states. The gray lines reflect logistic fits for each ACAM-J run, and the black line is the mean, with gray standard error shading. Afterglow is not plotted because main logistic models exclude afterglow.

We first inspected the average prevalence of each brain state over the course of the jhana meditation (Figure 3). To test for changes over the course of jhana states, we conducted mixed-effects logistic models, removing the afterglow. Indeed, state 1 significantly increased over time (Figure 4) (*B* = 0.092, *p*_*FDR*_ < 0.001), and State 2 decreased over time (Figure 5) (*B* = −0.15, *p*_*FDR*_ < 0.001). State 3 did not change over time (*p*_*FDR*_ > 0.05). This was consistent when examining *form* versus *formless* jhana; the prevalence of state 1 was higher during *formless* jhana (*B* = 0.38, *p_FDR_* < 0.001), and the prevalence of State 2 was lower during *formless* jhana (*B* = −0.61, *p_FDR_* < 0.001).

### Relationships to Phenomenology

**Figure 6.**
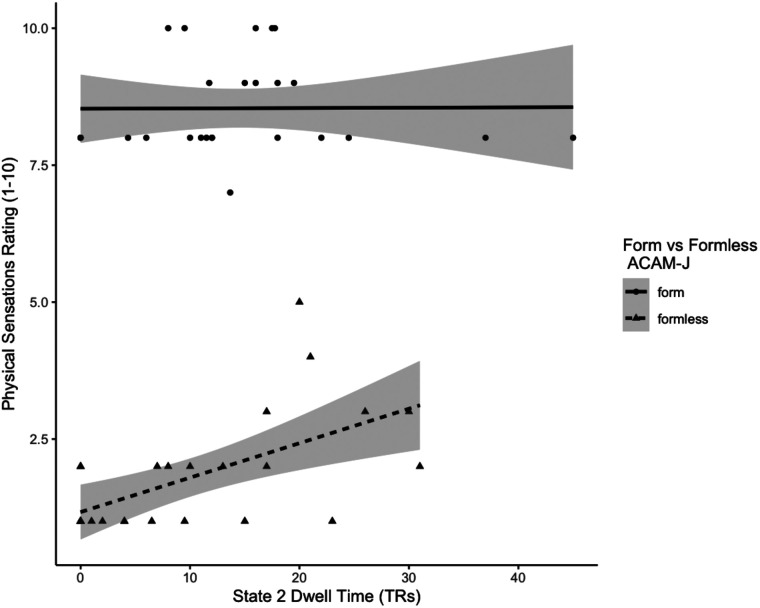
Self-report physical sensations versus dwell time in Brain State 2. Scatterplots are presented for ratings of physical sensations for 26 jhana runs versus dwell time in Brain State 2. On the *y*-axis, the ratings (1–10). On the *x*-axis, the dwell time, in number of TRs. Lines reflect smoothed linear fits, with standard error shading. The circle points and solid line reflect ratings during the form jhanas (ACAM-J1–J4), and the triangle points and dashed line reflect ratings during the formless jhanas (ACAM-J5–J8).

We first correlated the overall proportion of time spent in each brain state with global phenomenology—overall intensity, stability, and width of attention (Table 2). Width of attention was found to negatively correlate with proportion of time spent in Brain State 2 (*r*(24) = −0.43, *p* = 0.027). This finding was supported by noting that width of attention ratings increase over the jhana states (Supporting Information Figure S5), and Brain State 2 decreases in prevalence over jhana states. Canonical correlation analysis resulted in three factors, with a predominant factor that weighted Brain State 2 negatively and width of attention positively (*R*_*c*_^2^ = 0.59), and the variance explained by this factor was significant by Hotelling’s Trace (*p* = 0.03). Examining the relationship of local ratings—for specific jhana states as well as *form* versus *formless*, we found no relationships between bliss in J2, cool bliss in J3, equanimity in J4, narrative thought stream (*form* nor *formless*), intensity in any individual jhana state, width in any individual jhana state, and stability in any individual jhana state. However, we found a strong correlation between Brain State 2 dwell time in *formless* jhanas and physical sensations (*r*(24) = 0.60, *p* = 0.001) (Figure 6). The relationship between proportion of time (instead of dwell time) was in the same direction and trending toward significance (*r*(24) = 0.39, *p* = 0.05).

**Table 2.** Comparison of relationships between brain states and global phenomenology

**Phenomenology**	**State 1 proportion**	**State 2 proportion**	**State 3 proportion**
Width of attention	0.07	−0.43*	0.29
Intensity of attention	0.03	−0.18	0.11
Stability of attention	0.01	−0.03	0.02

Correlations were assessed between average ratings for each ACAM-J run and proportion of the brain state for each run.

* *p* < 0.05.

** *p* < 0.01.

*** *p* < 0.001.

### Clustering Trajectories

*K*-modes clustering revealed two optimal clusters. The first cluster consisted of 11 ACAM-J runs with prevalence of Brain State 1 increasing over the jhanas, and the other brain states decreased. In contrast, the second cluster, with the remaining 15 ACAM-J runs, exhibited consistently high levels of Brain State 3, while Brain State 1 was low and unchanging, and Brain State 2 decreased over the jhanas (Supporting Information Figure S6). Neurophenomenological analyses revealed that the second cluster (*M* = 8.40) had significantly higher width of attention (*t*(23.9) = 2.44, *p* = 0.022) compared with the first cluster (*M* = 8.00).

### Supplementary Whole-Brain Analyses

As a post hoc analysis, we conducted whole-brain clustering with all 526 regions. Results corroborated our targeted findings. There was higher static cortical connectivity during ACAM-J (Supporting Information Figure S7), differences in brain states (Supporting Information Figure S8) between ACAM-J and control runs, and more switches between brain states during ACAM-J (Supporting Information Table S2).

### Exploratory Practice Effects

As a post hoc analysis, we examined whether there were changes in brain states over the repeated ACAM-J runs. We found no relationship between Brain State 1 and run (*r*(24) = −0.11, *p* = 0.58), a significant negative relationship between Brain State 2 and run (*r*(24) = −0.40, *p* = 0.043), and a significant positive relationship between Brain State 3 and run (*r*(24) = 0.42, *p* = 0.033). Results were similar when examining days instead of runs.

## DISCUSSION

We conducted an intensive case study of ACAM-J, an advanced meditation that involves progressive changes in consciousness. ACAM-J involves initially focused attention, with concomitant experiences of bliss and joy, followed by increasingly wide and object-less attention, and reductions in verbal thought. Neuroscientific research on ACAM-J could provide mechanistic insight, and reveal the neurobiological endpoints of meditation (Chowdhury et al., 2023; Laukkonen et al., 2023; Sparby & Sacchet, 2024; van Lutterveld et al., 2024; Wright et al., 2023). Here, we conducted the first fMRI DFC analysis (Allen et al., 2014) of ACAM-J. In our study, we identified DFC changes related to sensory awareness, attention, and narrative self-referential processing.

We identified three predominant brain states: a DMN-anticorrelated brain state (1), a hyperconnected brain state (2), and a sparsely connected brain state (3). ACAM-J, compared with nonmeditative control conditions, showed higher prevalence of Brain States 1 and 2, more switches, and different transition probabilities. Brain State 3, sparsely connected and possibly an amalgamation of infrequent connectivity patterns (Mooneyham et al., 2017), was most prevalent during control conditions but remained consistent throughout jhanas. Therefore, we focused reporting on the DMN-anticorrelated brain state and the hyperconnected brain state.

### DMN-Anticorrelated Brain State

The prevalence of the DMN-anticorrelated brain state was higher in ACAM-J than control conditions. The control conditions used in this study were memory recall and mental counting, which likely both involve some form of verbalization. Furthermore, the DMN-anticorrelated brain state increased significantly over the course of the jhanas, and then returned to a low level during the follow-up period (afterglow). Moreover, the *formless* jhanas had significantly higher prevalence of the DMN-anticorrelated brain state than the *form* jhanas.

The DMN-anticorrelated brain state identified in this study specifically involved negative correlations between the left core DMN subsystem medial prefrontal cortex and the rest of the brain, as well as right control and right salience networks with the rest of the brain. Correlations between subcortex and brainstem with the cortex were low (excepting the periaqueductal gray and amygdala). Anticorrelations of the DMN with other brain networks have often been interpreted as supporting attention and the suppression of mind wandering (Bauer et al., 2020; Mooneyham et al., 2016, 2017). For example, DMN anticorrelations have been observed during cognitive tasks (Gao & Lin, 2012; Hellyer et al., 2014; Provost & Monchi, 2015). In addition, meditators who engaged in “silence” practice showed reduced connectivity between the DMN and language regions, perhaps reflecting the diminishment of spontaneous verbal thought (Tripathi et al., 2024). The left lateralization of the DMN anticorrelations we found seem to support this interpretation as widespread left hemisphere areas are involved in verbalization and language (Fedorenko et al., 2024). However, although the practitioner reported diminishing narrative processing throughout the jhanas, there was no correlation between reports of narrative processing and prevalence of this brain state.

It should be noted that while left core DMN regions like the MPFC showed anticorrelations with other networks, the other DMN regions did not. There is substantial evidence of DMN specialization (for review, see Kucyi et al., 2023). For example, as mentioned, frontal DMN regions are involved in mentalizing and linguistic thought (Andrews-Hanna & Grilli, 2021), and medial temporal lobe DMN may be involved in episodic memory, mental time travel, and mind wandering (Andrews-Hanna et al., 2010; Christoff et al., 2016; Ellamil et al., 2016; Kucyi & Davis, 2014). This suggests that the frontal DMN characteristics of this brain state may reflect suppression of linguistic thought more than “focused attention” per se. In the next section, we will point to some evidence that focused attention may be reflected more coarsely, in the hyperconnected state.

### Hyperconnected Brain State

The hyperconnected brain state involved elevated connectivity across many of the brain regions and networks included in our analysis. Hyperconnected brain states have been found across many DFC studies (Damaraju et al., 2014; de Lacy & Calhoun, 2018; Hutchison et al., 2013; Maillet et al., 2019; Mooneyham et al., 2017; Treves, Marusak, et al., 2024). We believe that there is evidence for a sensory processing interpretation of the hyperconnected brain state we found. First, there was meaningful structure in the elevated connectivity. Somatomotor networks, involved in sensory processing of body sensations, were particularly high in connectivity with the rest of the brain. Additionally, compared with Brain State 1, the thalamus and reward areas were correlated with cortical areas. The thalamus has a well-established role in gating sensory input to the cortex (Sherman, 2016; Wolff et al., 2021). Finally, the cerebellum was correlated with the dorsal attention network. The cerebellum forms afferent and efferent loops with cortical areas that may be involved in refining and shaping sensory predictions (D’Mello et al., 2020; Guell et al., 2018; Schmahmann et al., 2019). In summary, elevated somatomotor connectivity, thalamocortical connectivity, and cerebellocortical connectivity characterized Brain State 2, linking it to sensory processing mechanisms.

The prevalence of the hyperconnected brain state was higher in ACAM-J than control conditions, which putatively involve mental rehearsal and no sensory processing. Furthermore, the hyperconnected brain state decreased across jhanas. Sliced differently, the *formless* jhanas had significantly lower prevalence of the hyperconnected brain state than the *form* jhanas. Our practitioner reported using bodily sensations, that is, sensations of the breath, as objects to enter ACAM-J, but object orientation diminishes throughout the ACAM-J stages, especially in the *formless* jhanas. Finally, we observed negative correlations between self-reported width of attention and this brain state, and positive correlations between this brain state and physical sensations in the *formless* jhanas.

Similar phenomenological questions have rarely been applied with fMRI, but higher state mindfulness has been related to decreased DMN activations and decreased proportions of salience network anticorrelated brain states (Kim et al., 2019; Marusak et al., 2018). Attention to physical sensations (e.g., the breath) may result in widespread cortical deactivations (Farb et al., 2023). There is a rich literature on brain differences during focused attention in meditators and non-meditators (Fox et al., 2016; Ganesan et al., 2022); one salient finding is that meditators experience decreased dynamical brain complexity during focused attention meditation compared with rest (Escrichs et al., 2019). Our findings add to this literature by identifying a specific brain state related to self-reported narrow attention and attention to physical sensations.

Research outside of meditation suggests that hyperconnected brain states reflect heightened arousal and awareness (e.g., Treves, Marusak, et al., 2024). Interestingly, brain hyperconnectivity throughout many networks may be an emerging signature of the brain’s response to psychedelic drugs (Gattuso et al., 2023), as well as a difference present in individuals self-reporting psychosis symptoms (Barber et al., 2018). Psychedelic and psychotic experiences may both involve intense absorption and a suspension of organizing, rationalizing thought. Thus, the commonalities between these diverse states of consciousness underpinned by hyperconnectivity may be absorption. On the other hand, *hypo*-connectivity is sometimes associated with cognitive flexibility (Nomi et al., 2017). In keeping with this, the *hypo*-connected brain state in our analysis was less present in meditation than control conditions.

### Temporal Stability of Brain Function

ACAM-J meditators report increasingly stable attention over the course of jhanas, and a sense of deep stillness. The *formless* jhanas involve experiences of *boundless space* and *boundless consciousness*. These changes may be reflected in temporal stability of brain function. Interestingly, here, we found that ACAM-J involved more switches between brain states than control conditions, and this was not significantly different for *form* or *formless* jhanas. We did not find relationships between the number of switches and phenomenology. Research on anesthetics may provide an interesting parallel here—unconsciousness generally results in fewer dynamic brain switches and prolonged timescales (Barttfeld et al., 2015; Huang et al., 2018; Hutchison et al., 2014). ACAM-J states are clearly not inert, unconscious states. It is possible that temporal stability is found in some brain areas and networks and not others (Fong et al., 2019), and this could be assessed in future studies using seed-based dynamic conditional correlations (Lindquist et al., 2014).

### Limitations and Future Directions

Our main aim in this study was identifying brain states that were associated with changes in consciousness during ACAM-J meditation. Hypothetically, this could involve one-to-one mappings from each jhana to each brain state. This was found in the case of sleep stages—wake, slow-wave sleep (1–3), and REM were each associated primarily with one DFC state (Damaraju et al., 2020). Our study does not support a similar mapping. Instead, we found continuous trajectories of decreasing and increasing prevalence of the brain states. There was significant variability between trajectories, evidenced by multiple clusters of trajectories. We believe this reflects the parallel operation of the brain—the whole brain does not serially transition between activation or connectivity patterns, instead many brain connectivity patterns co-exist at the same times. Future work could examine a method called meta-state analysis, which allows for probabilistic assignment to brain states at each timepoint (Abrol et al., 2017; Miller et al., 2016). There might be consistent trajectories of meta-states during ACAM-J, if not specific connectivity patterns.

Early jhanas involve salient experiences of arising joy, bliss, and calm ease. We were surprised to find no relationship between self-report ratings of these experiences and the brain states. This is despite the finding that State 2 involves strong positive reward area connectivity (e.g., the nucleus accumbens) to the thalamus and cortex. Our previous study found ReHo of activations in reward regions correlated with self-report bliss. Activations may be more related to rewarding experiences than long-range connectivity between reward regions and the rest of the brain. This finding can also be interpreted in light of individual variability in ACAM-J practice. Some meditators may experience more significant feelings of bliss and joy during practice, and this could be represented in brain state characteristics. It is unclear whether the findings here generalize to other jhana practitioners. In general, work exploring these individual differences (perhaps related to school of practice) in addition to jhana development along a continuum of expertise would be worthwhile, while keeping in mind the requirement to collect reliable, high-quality, extensive data in order to capture individual differences.

On a related note, there was some evidence in this study of changes in ACAM-J over the course of scanning. Exploratory analysis showed increasing ACAM-J duration (especially early states) over days and runs, with changes in brain state prevalence within ACAM-J over time as well. These results do not bias our analyses as we performed within-jhana analyses using mixed-effects models with random slopes for run, but do suggest there may have been practice effects. Further work is necessary to characterize these changes.

Finally, the comparison of ACAM-J versus control conditions is limited by the necessity to combine across two control conditions for statistical power, and secondly because the meditator reported meditative states during the control conditions. There may be no true “baseline” in long-term meditators who practice extensively. Instead, long-term meditators may show extensive trait-level differences in terms of functional brain organization (Bauer et al., 2019; Kral et al., 2022; Sezer et al., 2022; Tripathi et al., 2024). It would be illuminating to examine whether the brain states uncovered in our analysis exist in control samples of meditation-naïve participants during natural rest (perhaps with “state” measurements of affect or attention). Alternatively, the brain states could be assayed in non-jhana meditation practitioners, with the aim of understanding commonalities between ACAM-J and other meditations.

### Summary

We conducted the first dynamic connectivity analysis of advanced meditation, extracting consistent, robust brain states that differentiated meditation from control conditions. The identified brain states changed significantly over the course of the advanced meditation, and reflected changes in attention, sensory awareness, and narrative self-referential processing. Future research could examine between-individual differences in the brain states.

## SUPPORTING INFORMATION

Supporting information for this article is available at https://doi.org/10.1162/netn_a_00432.

## AUTHOR CONTRIBUTIONS

Isaac N. Treves: Conceptualization; Formal analysis; Investigation; Methodology; Visualization; Writing – original draft; Writing – review & editing. Winson F. Z. Yang: Data curation; Investigation; Supervision; Visualization; Writing – review & editing. Terje Sparby: Writing – review & editing. Matthew D. Sacchet: Conceptualization; Funding acquisition; Project administration; Resources; Supervision; Visualization; Writing – review & editing.

## FUNDING INFORMATION

Matthew D. Sacchet, National Institute of Mental Health (https://dx.doi.org/10.13039/100000025), Award ID: R01MH125850. Matthew D. Sacchet, Dimension Giving Fund. Matthew D. Sacchet, Chade-Meng Tan of Buddhism.net and the Tan Teo Charitable Foundation. Matthew D. Sacchet, Ad Astra Chandaria Foundation (https://dx.doi.org/10.13039/501100022772). Matthew D. Sacchet, BIAL Foundation, Award ID: 099/2020.

## DATA AND CODE AVAILABILITY

Data may be requested and subjected to the Institutional Review Board’s approval. All code used in this study may be made available by request from the corresponding author.

## Supplementary Material





## TECHNICAL TERMS

Jhāna or Jhana: Literally, “meditation” in the Pali language, often refers to a series of absorptive meditation states.
ACAM: Advanced concentrative absorptive meditation (ACAM). A family of advanced meditation practices that are characterized by absorption.
Form jhānas: Initial four states of consciousness experienced during jhana, characterized by concentration on aspects of experience including bliss, joy, and equanimity.
Formless: Immaterial and not connected to ordinary perception and thought.
Formless jhānas: Four states of consciousness that follow the initial (form) jhānas, that include awareness of spaciousness and subtle consciousness.
DFC: Dynamic functional connectivity, time varying relationships between brain regions or networks.
Brain states: Prototypical dynamic functional connectivity patterns that can be extracted from dynamic functional patterns (often using clustering).
Proportion of a brain state: Patterns of brain activity that can be measured through various techniques, including electroencephalography (EEG) and functional magnetic resonance imaging (fMRI).
Average dwell time of a brain state: The average duration of the episodes of a brain state.
Episodes of a brain state: Time periods during which the brain exhibits an activity pattern or neural state.
Switches: The number of transitions between all brain states in a given time period.
Static connectivity: The statistical relationship (often Pearson’s correlation) between two resonance imaging (fMRI) signal timecourses extracted from two brain regions or networks.
