# Supplementary material for: Dynamic brain states underlying advanced concentrative absorption meditation: A 7-T fMRI-intensive case study
Source: Netw Neurosci. 2025 Mar 3;9(1):125–45. doi: 10.1162/netn_a_00432 (PMC11949543; doi:10.1162/netn_a_00432)
Supplement: Supplementary file 1 [file netn-9-1-125-s001.pdf]

Supplementary Information for

Dynamic brain states underlying advanced concentrative absorption meditation: A 7T fMRI intensive case study

Authors

Isaac N. Treves[1,2,3], Winson F.Z. Yang[1,2], Matthew D. Sacchet[1,2]*
(sacchetadmin@mgh.harvard.edu)

Supplementary Text

The value of case studies

There is currently increasing focus on big data in the field of fMRI. This is in part due to evidence that brain-behavior associations are small in effect, and require large samples (Marek et al., 2022; Winter et al., 2024). However, big data does not only encapsulate large sample sizes. Repeated, dense, sampling of individual subjects may be another way forward (Hung & Hsieh, 2022; Kucyi et al., 2021, 2024). Indeed, primate researchers have long employed this model – a typical experiment consists of training several primates in a specific task and collecting thousands of trials of data (Capitanio & Emborg, 2008; Roelfsema & Treue, 2014; Sarafyazd & Jazayeri, 2019). In primate research, there is no concern about generalizability – the research is understood to be a test of a specific model of how brains can be shaped and resulting cognitive function. This is also the paradigm in brain-computer interface research (He et al., 2020). We believe that case studies of meditators should be considered in the same way. Obviously, few individuals can volitionally experience radical changes in consciousness – but there is value in identifying the way their brains function for their inherent potential, i.e. "brain function can be shaped this way."

Post-hoc whole brain analyses

First, static connectivity corroborated the targeted results – there was higher cortical connectivity during *jhana* (**Figure S7**). We also found 5 states was the optimal solution for dynamic functional connectivity states, and we removed one noise-related state (**Figure S8).** Results from a comparison of control to *jhana* indicated that these brain states were sensitive to the manipulation. For example, just like the targeted analysis, we found more switches between brain states during *jhana* (M = 0.05 vs M = 0.04, *FDR-p* < 0.001). We did not further analyze these brain states because of concerns about spurious results and lack of cluster differentiation based on extremely high dimensionality.

Jhana	Mean (SD) time spent per run (in seconds)	Total time spent (in seconds)
1st Jhana (J1)	78.84 (35.41)	2128.60
2nd Jhana (J2)	91.94 (30.73)	2582.40
3rd Jhana (J3)	100.00 (71.15)	2699.90
4th Jhana (J4)	66.81 (24.60)	1803.80
5th Jhana (J5)	50.59 (13.76)	1365.90
6th-8th Jhana (J6-8)	123.84 (39.98)	3343.70

Supplementary Table 1: Duration of jhanas
Adapted from Yang et al. (2024a).

Measure	ACAM-J	Control	Difference	p_{FDR}
State1 Proportion	0.14	0.06	0.08	0.01**
State2 Proportion	0.27	0.08	0.19	< 0.001***
State3 Proportion	0.34	0.61	-0.28	< 0.001***
State4 Proportion	0.25	0.25	0	0.96
State1 Episodes	1.73	1	0.73	0.01**
State2 Episodes	3.15	1.75	1.4	< 0.001***
State3 Episodes	2.73	2.25	0.48	0.22
State4 Episodes	2.12	2.25	-0.13	0.61
State1 Dwell Time	10.54	7.13	3.41	0.07
State2 Dwell Time	14.62	5.79	8.83	< 0.001***
State3 Dwell Time	20.47	44.94	-24.46	< 0.001***
State4 Dwell Time	17.22	13.92	3.3	0.15
Proportion Switch	0.05	0.04	0.01	< 0.001***

Supplementary Table 2: Comparison of whole-brain dynamic measures between ACAM-J and control

Proportion reflects the proportion of TRs spent in a given state. Episodes refers to the number of unique periods spent in a given state. Dwell time reflects the average time spent in a brain state for a single episode. Proportion switch reflects how often (what proportion of TRs) involved a switch between brain states. * $p < 0.05$, ** $p < 0.01$, *** $p < 0.001$

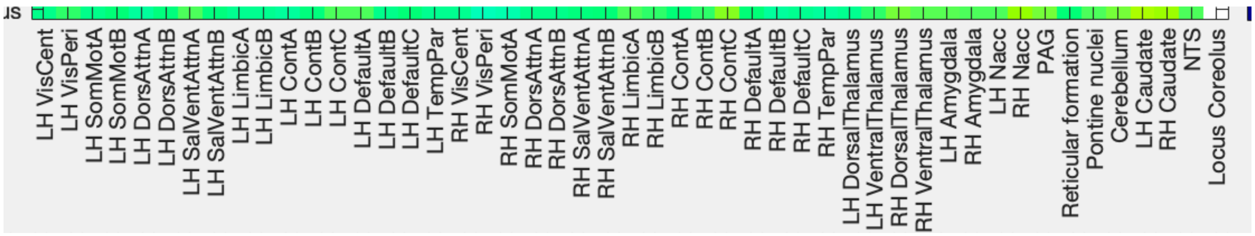

Supplementary Table 3: Brain networks and areas included

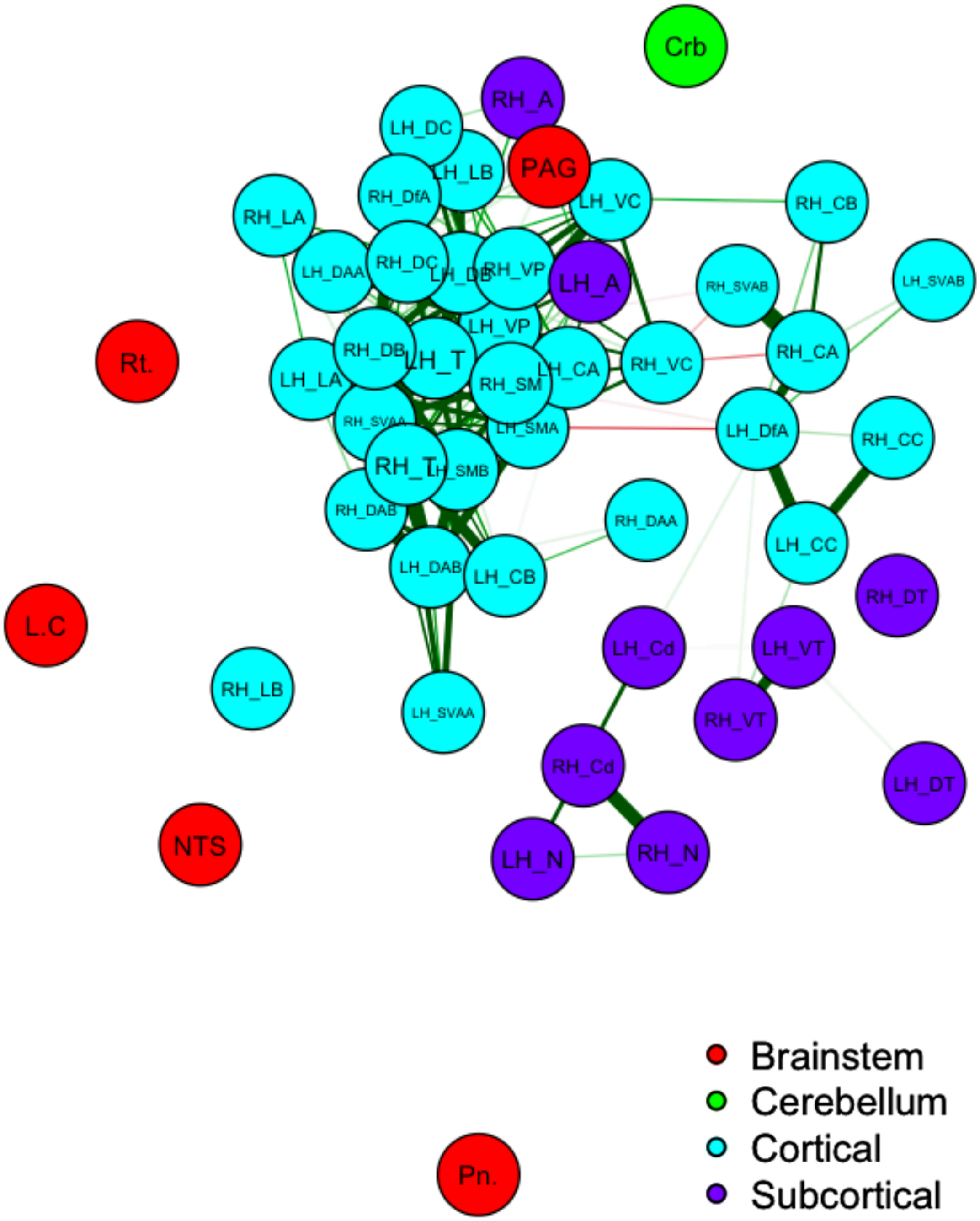

Figure S1: Brain State 1 connectivity network graph
Graph using *qgraph (spring layout),* with a correlation z-score threshold of 0.3. Nodes that are closer are more related. Anticorrelations are found between left DMN, right Control and right SAL networks and other cortical networks (blue). Caudate (Cd) and Nucleus Accumbens (NAcc) are correlated, in purple. Brainstem (red) and cerebellum regions (Crb, green) are uncorrelated. Amygdala (purple) and peri-acqueductal gray (red) are correlated to cortical networks.

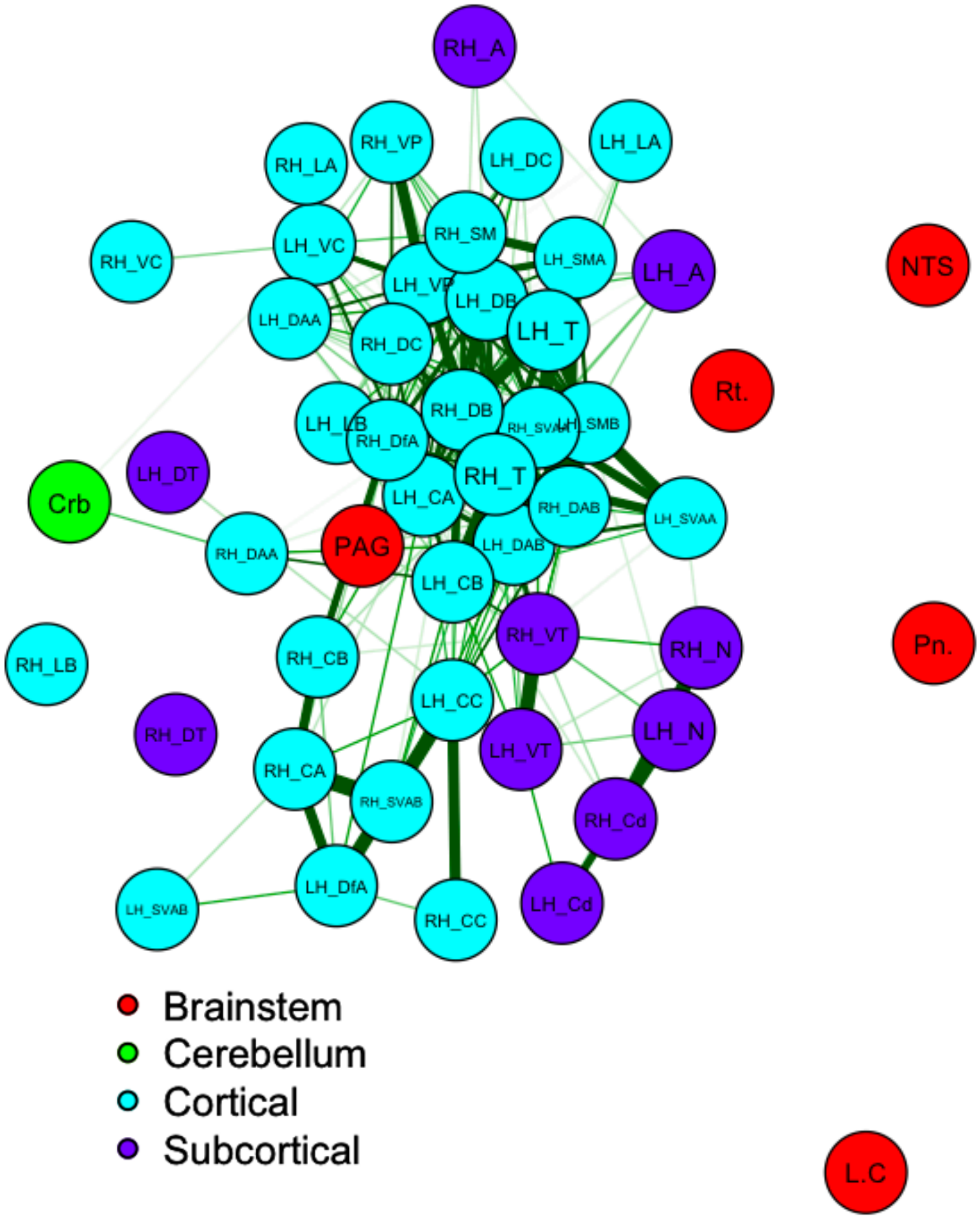

Figure S2:Brain State 2 connectivity network graph
Graph using *qgraph,* with a correlation z-score threshold of 0.3. Nodes that are closer are more related. Widespread positive correlations are found between cortical areas, subcortical areas, and cerebellum. The cerebellum is connected to the dorsal attention network. The left DMN is positively correlated with other cortical networks. The caudate and nucleus accumbens (purple) are now connected to cortical areas. The amygdala and periacqueductal gray are positively correlated with cortical areas.

Brain State 1 minus Brain State 2

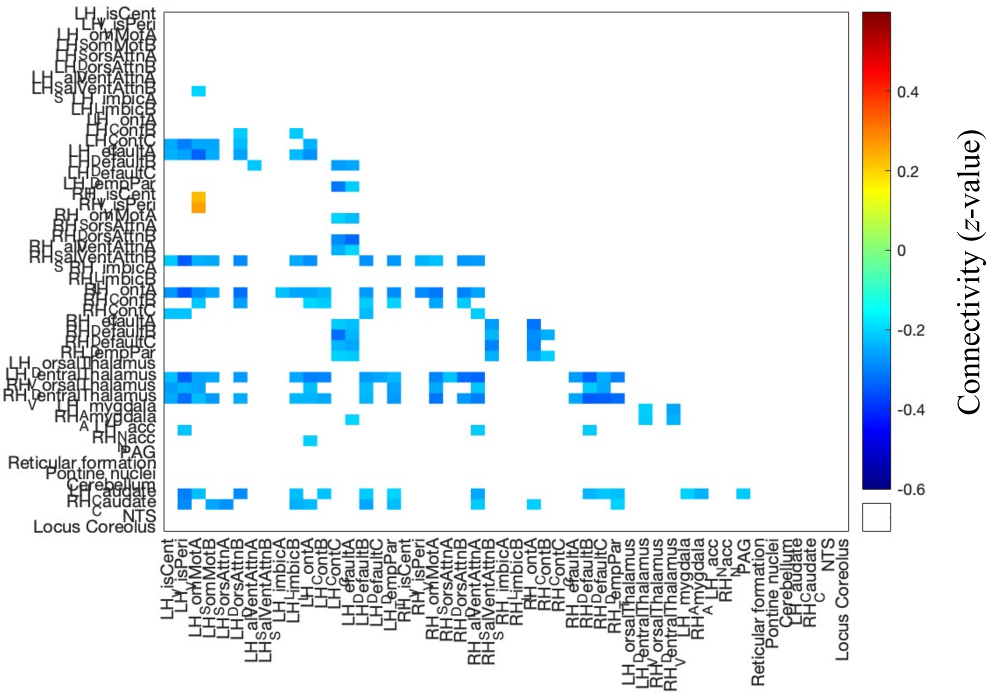

Figure S3: Brain States 1 and 2 compared.
Difference between connectivity in Brain State 1 and connectivity in Brain State 2, with an absolute threshold of 0.2. Brain State 1 has decreased connectivity in thalamus and caudate, left hemisphere default mode network, control networks, and right hemisphere salience networks.

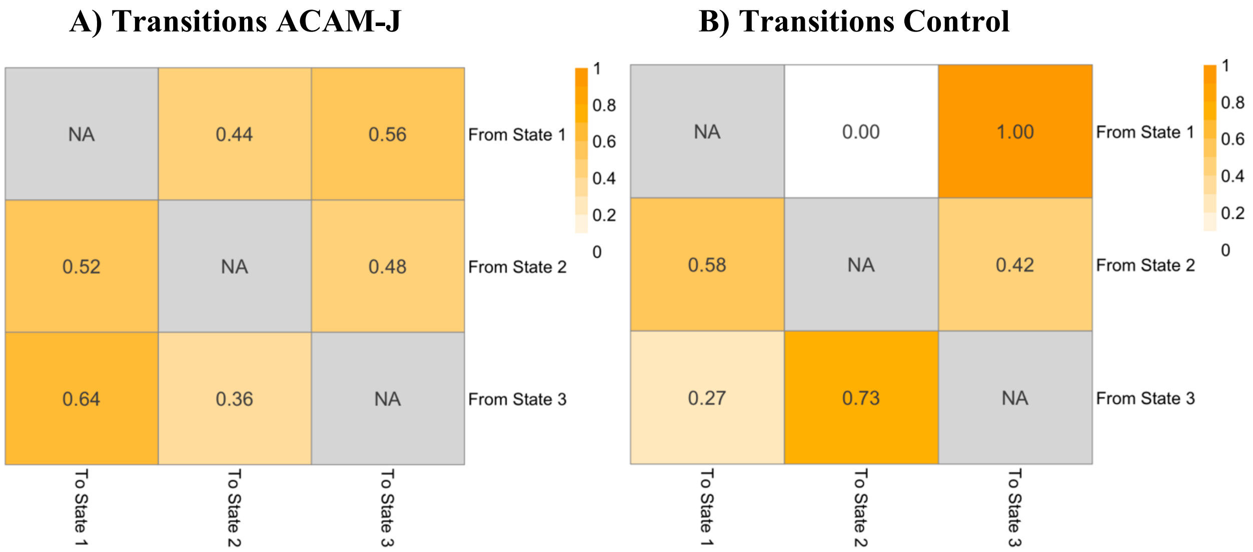

Figure S4: Transition matrices between the three brain states for ACAM-J vs control. Deeper orange shade reflects increased probability. We have normalized based on out-of-state transitions, as most TRs involve remaining in a given state (NAs). A) In ACAM-J, State 1 transitions to State 2 and 3, and state 3 transitions primarily to state 1. B) In control conditions, State 1 primarily transitions to state 3 and state 3 transitions primarily to state 2.

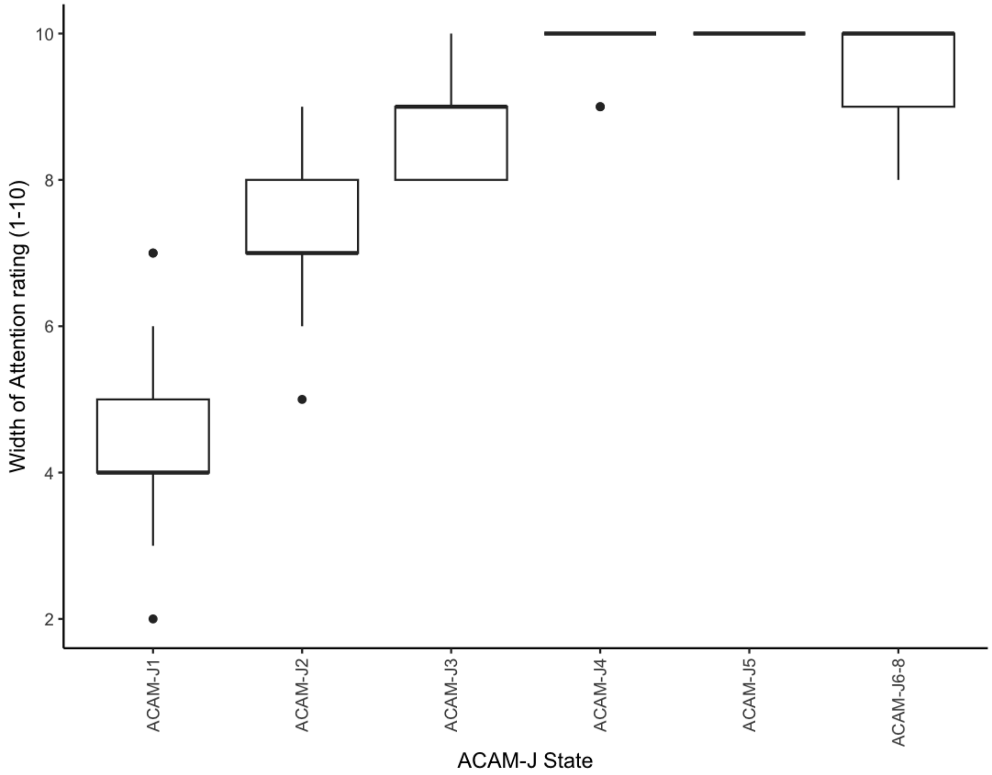

Figure S5: Width of attention ratings for jhanas
Width of attention ratings for each advanced concentrative absorptive meditation – jhana (ACAM-J) stage.

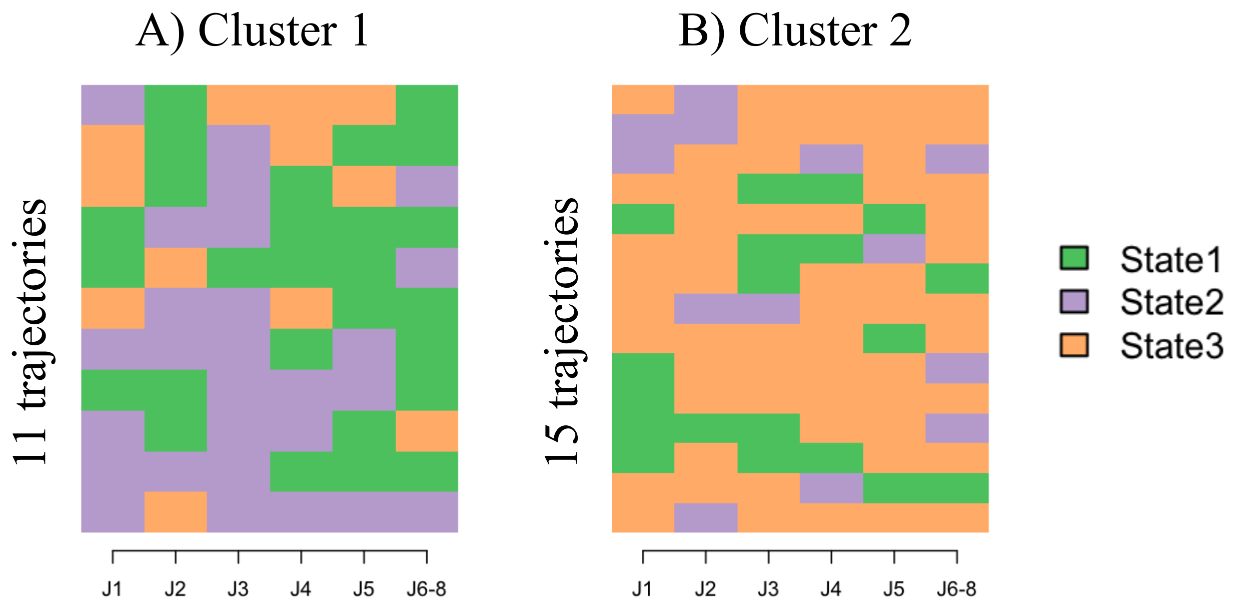

Figure S6: Clustering of brain state trajectories during ACAM-J.
Heatmaps show the most common brain state for each jhana and run. A) Cluster 1, consisting of 11 trajectories (y-axis), mostly consisting of Brain State 2 decreasing and Brain State 1 increasing. B) Cluster 2, consisting of 15 trajectories (y-axis), mostly consisting of Brain State 3.

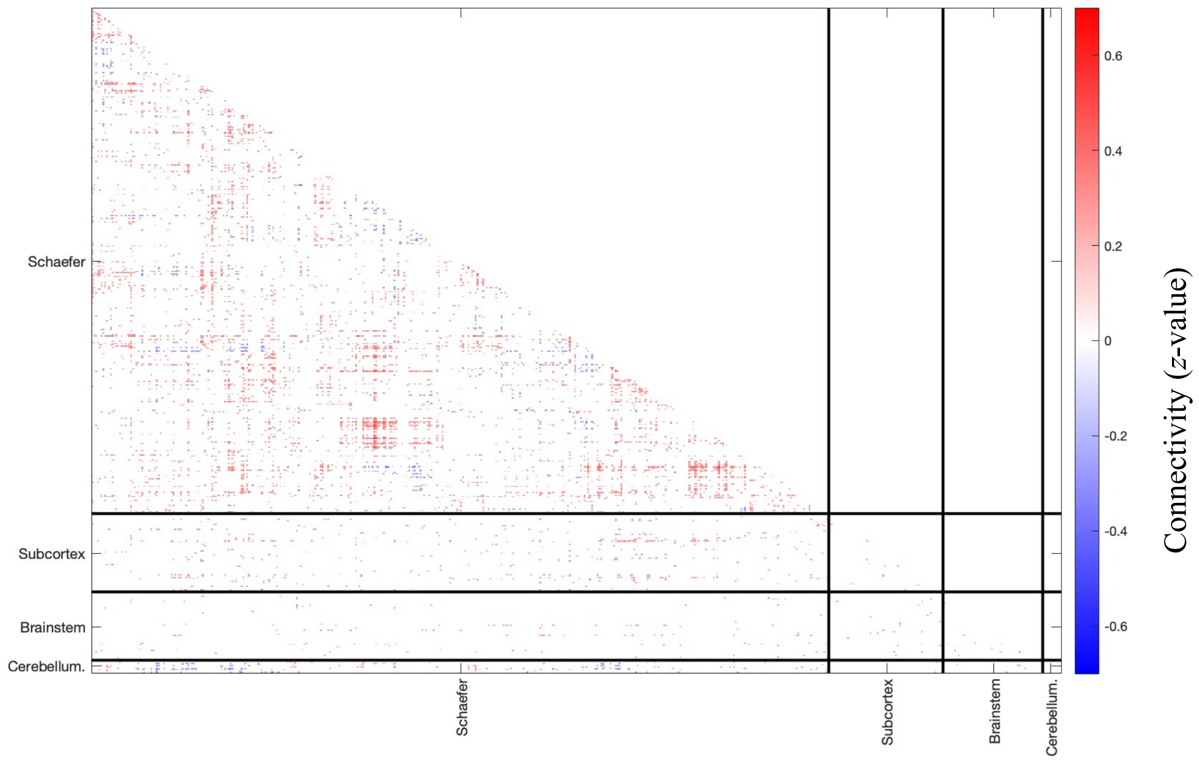

Figure S7: ACAM-J minus control static functional connectivity for whole-brain analysis. Whole-brain (526 ROIs) connectivity is plotted at an absolute threshold of 0.2. In general, more positive correlations between cortical regions are found during ACAM-J than control, as was found in targeted analysis. Additionally, cortico-cerebellar connectivity was lower in ACAM-J.

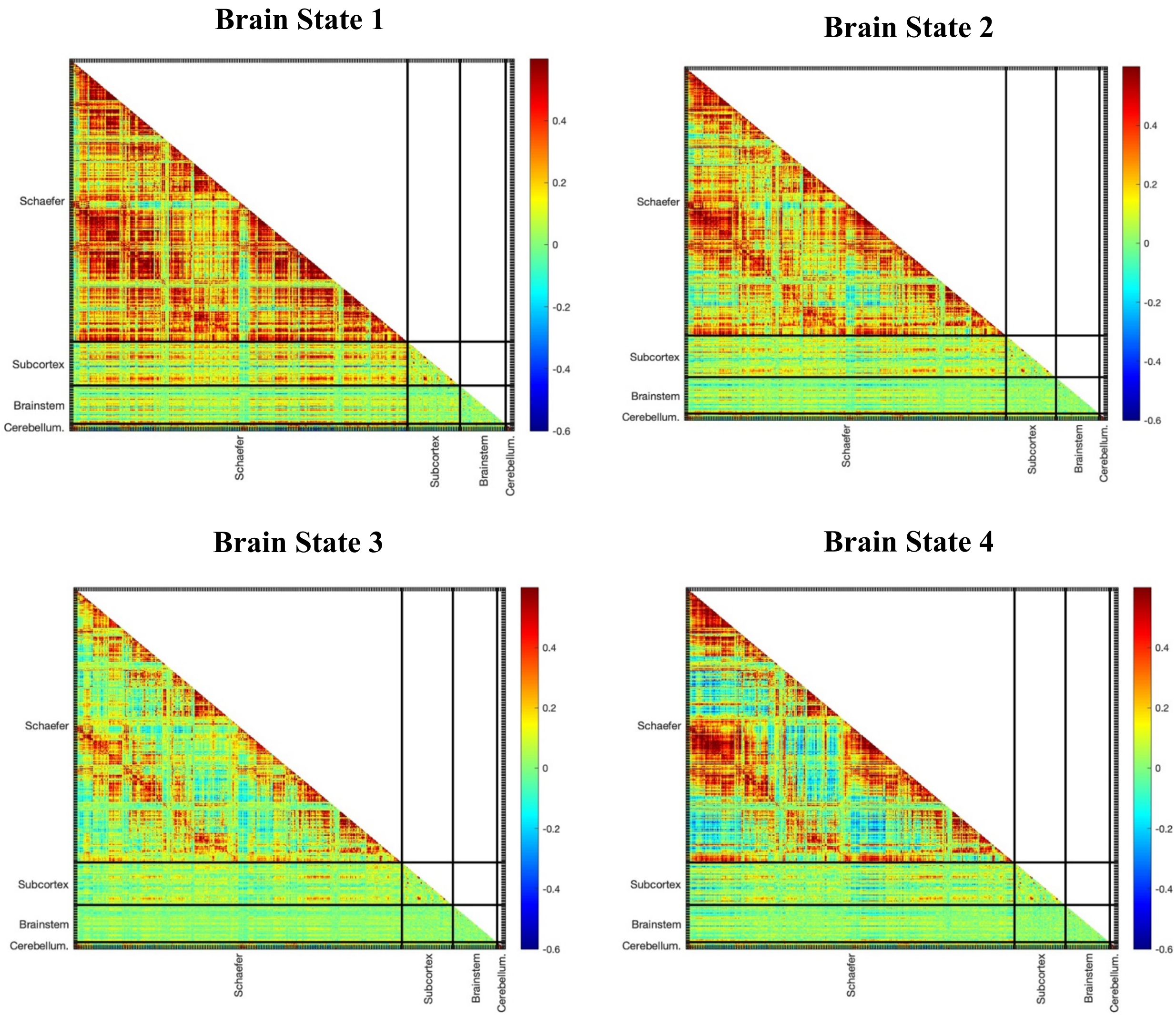

Figure S8: Dynamic functional connectivity states from whole-brain analysis.
Color indicates *z*-scored connectivity.
